# Access to Daylight at Home Improves Circadian Alignment, Sleep, and Mental Health in Healthy Adults: A Crossover Study

**DOI:** 10.3390/ijerph18199980

**Published:** 2021-09-23

**Authors:** Rohan Nagare, May Woo, Piers MacNaughton, Barbara Plitnick, Brandon Tinianov, Mariana Figueiro

**Affiliations:** 1 Light and Health Research Center, Department of Population Health, Science and Policy, Icahn School of Medicine at Mount Sinai, New York, NY 10029, USA; barbara.plitnick@mountsinai.org (B.P.); mariana.figueiro@mountsinai.org (M.F.); 2 View, Inc., Milpitas, CA 95035, USA; may.woo@view.com (M.W.); piers.macnaughton@view.com (P.M.); brandon.tinianov@view.com (B.T.); 3 Department of Environmental Health, Harvard T.H. Chan School of Public Health, Boston, MA 02115, USA

**Keywords:** daylight, circadian light, lighting for indoor environments, electrochromic glass, blinds, sleep, melatonin, healthy building, circadian alignment, residential lighting

## Abstract

As the primary environmental cue for the body’s master biological clock, light–dark patterns are key for circadian alignment and are ultimately fundamental to multiple dimensions of health including sleep and mental health. Although daylight provides the proper qualities of light for promoting circadian alignment, our modern indoor lifestyles offer fewer opportunities for adequate daylight exposure. This field study explores how increasing circadian-effective light in residences affects circadian phase, sleep, vitality, and mental health. In this crossover study, 20 residents spent one week in their apartments with electrochromic glass windows and another week with functionally standard windows with blinds. Calibrated light sensors revealed higher daytime circadian-effective light levels with the electrochromic glass windows, and participants exhibited consistent melatonin onset, a 22-min earlier sleep onset, and higher sleep regularity. In the blinds condition, participants exhibited a 15-min delay in dim light melatonin onset, a delay in subjective vitality throughout the day, and an overall lower positive affect. This study demonstrates the impact of daytime lighting on the physiological, behavioral, and subjective measures of circadian health in a real-world environment and stresses the importance of designing buildings that optimize daylight for human health and wellbeing.

## 1. Introduction

Circadian rhythms are the platform for all biology and are observed in several physiological, psychological and behavioral processes, in particular the sleep–wake patterns [1]. In humans, endogenous circadian rhythms are generated and regulated by the body’s master biological clock, operating within the hypothalamic suprachiasmatic nuclei (SCN) [2]. In the absence of any zeitgebers (i.e., external time cues), this master biological clock free-runs with an average period of approximately 24.2 h. The 24 h pattern of light and dark reaching the retina entrains (or synchronizes) the timing of the biological clock to the 24 h solar day.

The master clock promotes alertness during the day and sleepiness at night in all diurnal species, predominantly through the regulation of hormones. The rise of melatonin in the hours preceding sleep triggers sleep onset. Conversely, cortisol increases in the hours prior to waking and informs the body of the night–day transition (or between inactivity and activity). These endogenous signals generated by the master clock oppose the homeostatic sleep drive to promote daytime alertness and nighttime sleepiness [3]. Specifically, stable and high daytime levels of alertness and consolidated nighttime sleep are maintained when the phase relationship between the internal circadian timing system and the sleep-wake cycle is aligned.

As the primary environmental cue for the body’s master biological clock, light has been shown to impact sleep, mood, performance, alertness, quality of life, and overall health in various populations [4,5]. Optimizing light for the promotion of circadian alignment in architectural settings involves ensuring the proper levels, timing, duration, and spectra of light that occupants are exposed to throughout the day while minimizing their exposure during the evening, prior to bedtime. Daylight is an ideal light source for promoting circadian alignment due to its natural 24 h cycle, high light levels, and spectral power distribution which provides the proper circadian stimulus at all times of day.

Previous research monitoring 109 participants across seven consecutive days demonstrated that high levels of circadian-effective light during the entire day were associated with better alignment between light–dark and rest–activity patterns [6], better sleep quality, and lower depression scores. In a 2014 study involving 49 office workers, Boubekri et al. [7] reported superior sleep quality, increased sleep duration, and increased physical activity in participants working in windowed environments with more daylight access. In a recent survey involving 593 middle-aged adults, an increase in daily outdoor time and brighter indoor environments was associated with significantly reduced feelings of anxiety and depression and fewer sleep disturbances [8]. A recent study across 12 European countries also found that daylighting conditions affect schoolchildren’s performance [9].

Although these and other studies clearly demonstrate the benefits of daylight, modern lifestyles (largely spent indoors) afford fewer opportunities for receiving adequate daylight exposure. Enabling daylight effectively in indoor environments therefore presents a powerful opportunity for promoting improved nighttime sleep and better mood in humans, while also reducing energy use from electric lighting. While abundant daylight access is the design intention for most modern buildings, in practice windows are often covered by blinds to control for unwanted heat and glare, effectively limiting daylight access and impeding proper exposure to daytime circadian-effective light [10]. This issue is compounded by behavioral tendencies, wherein an occupant is unlikely to change the position of the blinds, even on cloudy days, once they have been adjusted to block direct sunlight, tendencies that have been demonstrated in both office and residential settings [11]. Electric lighting is a modest substitute for daylight, as it is unable to match the light levels of sunlight in commercial applications and has a fixed correlated color temperature, while daylight varies in light level and spectral power distribution throughout the day [12].

One modern technology which facilitates daylight in indoor environments, while also addressing the challenges described above, is electrochromic (EC) glass. EC glass works by applying a low-voltage electric current to a thin electrochromic coating embedded within windows layers, which changes the window from clear to tinted. EC glass can also automatically tint in response to the presence and timing of direct solar radiation, effectively eliminating the need for occlusion by blinds or shades since it mitigates dynamic glare and heat conditions. EC glass also allows for more penetration of short-wavelength light, which is optimal for affecting the circadian system. Due to its contributions toward designing smarter, more sustainable, and healthier buildings, EC glass has become adopted at scale in a variety of markets including offices, healthcare facilities, residential buildings, and airports, with projects greater than one million square feet and totaling approximately 100 million square feet of adoption worldwide. This technology has recently demonstrated improvements to occupant sleep duration, cognitive performance, and environmental satisfaction while mitigating physical discomfort symptoms such as eyestrain and headaches [13]. The current study is intended to build upon this research by focusing on the non-visual effects of indoor daylight levels as delivered by EC glass technology relative to the use of conventional windows and blinds. Our theoretical construct was that daylight, if enabled effectively in indoor environments, will promote the circadian alignment of the endogenous rhythms (e.g., melatonin rhythm characterized by consistency of dim-light melatonin onset) with the local day–night cycle, leading to improved nighttime sleep and daytime feelings of vitality.

The aim of this four-week, within-subjects, crossover design field study was to investigate the effectiveness of EC glass designed to reduce glare and increase circadian-effective light indoors on measures of circadian phase, sleep, vitality, and mental health in a residential environment. Due to the COVID-19 pandemic, participants spent the great majority of the study period in their own apartment units and experienced two experimental conditions in randomized order: (1) one eight-day intervention period with functionally standard (i.e., clear) windows with blinds partially drawn and (2) one eight-day intervention period with functioning EC glass windows. A six-day baseline period preceded the participants’ first intervention period, and a six-day washout period separated the two interventions. We hypothesized that the EC glass condition would increase participants’ daylight access and thereby lead to both stronger alignment of rest-activity patterns with the day–night cycle and improved objective and subjective sleep and mental health outcomes.

## 2. Materials and Methods

### 2.1. Study Population

Twenty residents were recruited from an approximately 400-unit luxury apartment complex (composed of two towers) in Reston, Virginia, via informational flyers posted in the apartment buildings and emails sent to building residents. Study eligibility was restricted to individuals over the age of 21 who were staying or working at home full-time during the study period, and who did not have an active diagnosis of insomnia, sleep apnea, seasonal or chronic depression, claustrophobia, or schizophrenia. Participants were required to maintain a regular sleep–wake schedule and not use oral melatonin or sleep medication. Participants were not informed of the nature of the experimental conditions, nor of the study’s hypotheses, in any of the informational materials or study correspondence.

Upon enrollment, the participants completed a baseline survey (administered via Qualtrics [Provo, UT, USA]) which collected their basic demographic data, general health status (adapted from the Medical Outcomes Study [MOS] 36-Item Short-Form Health Survey [SF-36] [14]), and chronotype as assessed via the Munich ChronoType Questionnaire (MCTQ) [15] (Table 1). The study protocol was reviewed and approved by the Icahn School of Medicine at Mount Sinai (IRB approval #20-01654) and Rensselaer Polytechnic Institute (IRB approval #1943) institutional review boards. The investigations were carried out following the rules of the Declaration of Helsinki of 1975 and subsequently established ethical standards [16,17]. All participants signed informed consent documents and received a nominal stipend for participation in the study.

### 2.2. Experimental Conditions

The experimental conditions were delivered via the windows of the participants’ apartments, which ranged in size from one to two-bedroom units and ranged in elevation from ground-level to the 14th floor. Among the 20 participants, eight were cohabitants; therefore, the experimental conditions comprised of a total of 16 unique apartment units. Twelve units had southeast-facing façades and four had northwest-facing façades. During the active intervention periods, participants were asked to predominantly remain inside their apartments during the daytime hours, and their compliance was tracked via daily surveys.

All participants were exposed to the two experimental conditions over the course of the study’s two intervention periods. In one of these, the EC Glass condition, the EC glazing of participants’ windows automatically tinted or cleared based on the presence and timing of direct solar radiation on the building’s façade or based on participant’s own control via a proprietary mobile app. The four tint states ranged from a visible transmittance of 58% down to 0.5%, becoming increasingly dark and filtering out longer wavelengths of light while admitting shorter wavelengths of light at higher tint states. The spectral distributions of the four tint states are described further in Supplementary Figure S1. For the second Blinds condition, the EC windows were set to their clearest state, effectively functioning as standard windows, and participants were free to adjust their blinds so long as they were at least pulled halfway down.

**Figure 1:**
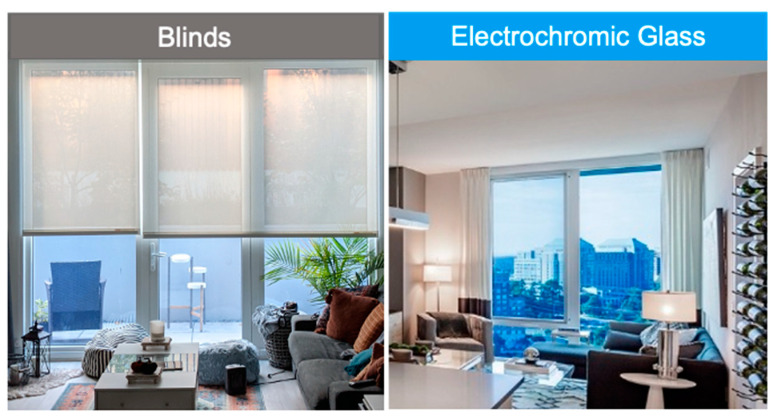
Representative images of the blinds and EC glass study conditions.

The order of conditions was randomly assigned between two participant groups (see Section 2.4) and each intervention period was preceded by a baseline/washout period, during which participants were free to use their EC glass windows and blinds as they would typically have done prior to commencement of the study. Representative images of the two conditions are shown in Figure 1.

### 2.3. Study Outcomes

#### 2.3.1. Environmental Conditions

Environmental conditions were monitored throughout the study using commercially available Awair Omni (San Francisco, CA, USA) environmental sensors installed on each apartment’s living room wall adjacent to the window. These devices measured illuminance (lux), temperature (degrees Fahrenheit), relative humidity (percent), air quality (CO_2_ in ppm, PM2.5 in µg/m^3^), and noise levels (decibels) with a 5-min resolution, and data were digitally obtained from the Awair web dashboard in real-time. Mean daytime illuminance (lux) was calculated for each condition using data collected from sunrise to sunset (approximately 7 AM to 5 PM) (Figure S2; further details are provided in the Supplementary Material).

#### 2.3.2. Objective Sleep and Rest-Activity Patterns

Participants’ sleep patterns were recorded using wrist-worn actigraphs (Actiwatch Spectrum Plus, Philips North America Corporation, Cambridge, MA, USA) throughout the entire study. They also wore a personal circadian light monitor called a Daysimeter [18] as a pendant (i.e., approximately at chest level) from waketime to bedtime to record their rest-activity patterns and personal light exposures during the intervention periods only.

Actigraph data were post-processed using the device’s proprietary software (Actiware 6.0, Philips North America Corporation, Cambridge, MA, USA) to obtain activity data throughout the day and objective sleep measures for each sleep period: sleep onset, sleep duration, sleep onset latency, and sleep efficiency. Compliance was tracked by the wear sensor on the back of the actigraph and observations marked as non-compliant were excluded. Actigraph data were obtained for 89% of the total number of participant observation-days across the entire study period and 85% across the two intervention periods. Sleep duration, latency, efficiency, and sleep onset analyses were restricted to weekday, non-sampling nights (nights when biospecimens were not collected [see below]) across intervention periods. Weekend nights (i.e., Friday and Saturday nights) were excluded due to greater variability in social activity schedules, and sampling nights were excluded as the biospecimen collection necessitated participants to stay up 1.5 h beyond their typical bedtime and, therefore, artificially alter their sleep schedules. Within-subjects comparisons of mean sleep metrics across these weekday, non-sampling nights followed the procedures described in Section 2.5.

To assess the impact of time spent in each intervention, the sleep outcome means at the start (i.e., the first two non-sampling weekday nights) and end (i.e., the last two non-sampling weekday nights) of each intervention period were plotted. Sleep regularity, as measured using the Sleep Regularity Index (SRI) [19], was derived from the actigraph data. Briefly, SRI ranges from zero to 100 and is a measure of the minute-by-minute likelihood that any two timepoints 24 h apart are in the same sleep or wake state. As circadian disturbances may manifest in oversleeping or shifted sleep timing on the weekends, SRI was calculated as the mean across all non-sampling nights within the intervention (that is, both weekend and weekday nights). Additionally, SRI was calculated for the start (first two non-sampling weekday nights) and end (last two non-sampling weekday nights) of the intervention periods to evaluate any association with duration in intervention.

Daysimeter data, collected for the rest-activity and personal light exposure analyses (sampling time of 180 s), were processed using MatLab (Version R2020b, The MathWorks, Inc., Natick, MA, USA) to perform phasor analysis as specified in Miller et al. [20]. Mean daytime (approximately 7 AM to 5 PM) circadian stimulus (CS) [21,22] in each experimental condition was derived using data collected on all days within each intervention period. Calculations of mean daytime melanopic lux were derived from the Daysimeter data by spectral weighting of the calibrated RGB sensor data. Circadian light (CL_A_) [23] calculations from the Daysimeter data were merged with activity profiles from the actigraph to derive the 24 h phasor profiles necessary for quantifying circadian alignment of activity to light. Five participants failed to meet the threshold compliance criteria for three full days of Daysimeter data and thus were excluded from the phasor analyses.

Briefly, phasor analysis is based upon circular correlations between the periodic changes in light and activity time series to gauge how the correlation changes as a function of the phase difference between the two series. The circular correlation function was then decomposed into its temporal frequencies and phase angles using Fourier analysis techniques to generate circadian phase attributes of phasor magnitude and phasor angle. Phasor magnitude is a metric for behavioral circadian alignment/disruption, wherein the greater the magnitude (range: 0–0.7), the greater the level of behavioral circadian alignment of activity to light. The phasor angle (range: +12 to −12 h) reflects the phase relationship between the periodic light–dark exposure pattern and the periodic activity–rest pattern in the correlations. (A more detailed description of phasor magnitude, phasor angle, and the Daysimeter are provided in the Supplementary Material).

#### 2.3.3. Biospecimen Collection for Dim Light Melatonin Onset (DLMO) Analysis

On the first and last days of each intervention period, participants collected 10 saliva samples during the evening hours for DLMO analysis, under a researcher’s supervision, either in person or remotely via video conferencing. At minimum, one researcher at a time was actively supervising samples collected via video conferencing at all times. This involved instructing, timing, and watching participants during each step of the sampling procedure including ensuring that each sample was placed into their freezer immediately after collection. Samples (1 mL) were collected using the Salivette system (Sarstedt, Newton, NC, USA), wherein participants chew on a plain (not citric-impregnated) cotton cylinder for approximately 1 min. Sample collection began 3 h before the participant’s customary bedtime and continued every 30 min thereafter until 1.5 h past their customary bedtime. (For example, for someone who typically goes to bed at 11 PM sample collection would occur from 8 PM to 12:30 AM). Sampling times were scheduled at the start of the study based on each participant’s MCTQ score obtained upon enrollment in the study (see Section 2.1, Table 1), and compliance with these times was ensured by distributing email calendar invites and scheduled SMS text reminders 5 min prior to each sample with the links to the supervision video call. Additional reminder SMS messages were sent to participants who were 10 min late or more to the scheduled sampling session. To maintain dim light during data collection, participants were asked to lower the ambient light levels in their apartments and wear orange glasses to control the amount of light they were receiving. Participants were asked to rinse their mouths and brush their teeth (without toothpaste) before any samples that occurred following meals or snacks.

Following collection, each sample was immediately frozen and the day’s batch was shipped to the Mount Sinai Light and Health Research Center, where they were centrifuged (for 5 min at 1000× *g*) and radioimmunoassayed (Catalog number 79-MEHLU-R100, Direct Melatonin RIA, ALPCO, Salem, NH, USA). Masked quality control samples (10%) were included. The sensitivity of the saliva assay for melatonin radioimmunoassay was 0.7 pg/mL and the intra and inter-assay coefficients of variability (CVs) were 12.1% and 13.2%, respectively. None of the study participants reported any issue with providing the saliva samples and 100% of samples were collected and analyzed.

DLMO thresholds were calculated using a technique published in the literature, which takes the average of the five continuous lowest melatonin levels plus 15% of the five continuous highest melatonin levels (“5 H/5 L”) [24]. The time after which melatonin levels of at least three samples remain above this threshold value is determined to be the DLMO time.

#### 2.3.4. Surveys

The participants responded to three separate series of surveys that were administered at varying intervals over the course of the study. First, on the first (Days 7 and 21) and last (Days 14 and 28) days of each intervention period, participants completed a survey assessing their sleep-related impairments, mental health, and mood via six Patient-Reported Outcomes Measurement Information System (PROMIS) instruments [25,26]:

1. Sleep disturbance 4a (SD4a), which measures perceptions of sleep quality, depth, and restoration (i.e., Sleep quality rating, with higher score as poorer quality) using four items;
2. Sleep-related impairment 8a (SRI8a), which measures perceptions of alertness, sleepiness during waking hours, and associated functional impairments (e.g., “I had a hard time getting things done today because I was sleepy”) using eight items;
3. Anxiety 4a (A4a), which measures self-reported fear, panic, anxious misery, and tension (e.g., “My worries overwhelmed me”) using four items;
4. Psychological stress 4a (PS4a), which measures feelings of being overwhelmed or lacking control (e.g., “I felt stressed”) using four items;
5. Depression 4a (D4a), which measures negative mood and views of self-worth (e.g., ”I felt depressed”) using four items; and
6. Positive affect 15a (PA15a), which measures momentary experiences such as pleasure, joy, pride and engagement (e.g., “I felt attentive”) using 15 items.

PROMIS instruments have demonstrated high internal consistency in several populations (Chronbach’s alpha values above 0.80) [26,27] and high content validity, indicating that the domain names represent the item bank content [28,29].

All PROMIS instruments were scored according to each instrument’s manual to produce composite T-scores [30], where a higher score indicates a greater degree of the concept being measured (e.g., higher scores indicate greater sleep disturbance for SD4a and greater positive affect for PA15a). T-scores have a mean of 50 and standard deviation of 10 in the referent general population, and cutoffs vary slightly depending on the instrument. For sleep disturbance and sleep related impairment, scores below 55 indicate normal and scores of approximately 55, 59 and 65 are lower thresholds for mild, moderate, and severe, respectively. For the anxiety and depression scales, scores below 55 indicate normal and scores of approximately 55, 60 and 70 are the lower thresholds for mild, moderate and severe, respectively [30]. Thus, except for positive affect (PA15a), lower PROMIS T-scores represent improved outcomes. The weekly survey also collected participants’ subjective assessments of indoor environmental satisfaction over the week for temperature, air quality, humidity, noise, glare, and daylight. Overall compliance with the weekly survey was 100%.

For the second survey, administered during the active intervention weeks only, participants completed a survey every evening at approximately 6:00–8:00 PM that collected information on the following lifestyle factors: (1) amount and timing of caffeine and alcohol consumption; (2) amount of time spent watching TV or using a computer in the evening; and (3) over-the-counter or prescription medication use. One question from each PROMIS instrument was also included in the daily survey, with its selection being based on the dimension of interest: sleep quality (SD4a); sleep-related impairment (SRI8a); anxiety (A4a); stress (PS4a); depression (D4a); and positive affect (PA15a). These questions were analyzed as the percent difference in raw scores between conditions (reported on a scale of 1–5 points, again with higher scores indicating greater a degree of the concept being measured). Data from all intervention days, including weekends and sampling days, were included. Overall compliance with the daily survey was 100%.

For the third survey, administered on the first (Days 7 and 21) and last (Days 14 and 28) days of each intervention period, participants also completed a Subjective Vitality Scale (SVS) [31] instrument every 4 h on a schedule aligned with their MCTQ score (e.g., 7 AM, 11 AM, 3 PM, 7 PM, 11 PM and 7 AM the following morning for someone with a MCTQ score of 3 [normal]). The SVS prompts respondents to rate their level of agreement with six positive statements of vitality (e.g., “feeling alive”, “awake”, “bursting with energy”) and one negative statement (“not feeling energetic“) each on a seven-point Likert scale. A composite vitality score was derived by taking the mean of the scales for the six positive statements, as done in previous studies [32,33]. Overall compliance with the SVS survey was 99%.

### 2.4. Study Protocol

The two experimental conditions employed in this within-subjects, crossover-design field study used EC glass windows present in the study site’s two buildings. The four-week protocol was conducted in November through December 2020. The participants were divided into two groups. Group 1 (10 participants, mean age = 28 years [SD 4.9]) underwent the EC Glass condition first and the Blinds condition second. Group 2 (10 participants, mean age = 42 years [SD 15.2]) underwent the Blinds condition first and the EC Glass condition second.

**Figure 2:**
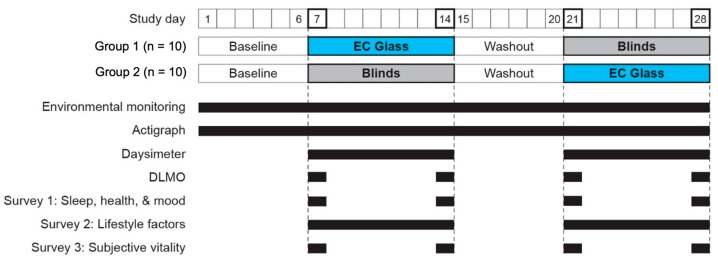
The study protocol, showing the schedule for delivery of the two experimental conditions and data collection for environmental conditions, objective sleep (actigraph), rest-activity and personal light exposures (Daysimeter), biospecimen collection for DLMO, and the three surveys. DLMO and subjective vitality (SVS data) were collected every 4 h on the first and last days of the intervention periods (shown in bold).

Participants maintained their daily routines in their own apartments throughout the course of the study, which was composed of two six-day baseline/washout periods, each followed by an eight-day period exposing participants to one of the two experimental conditions (see Section 2.2). During the intervention periods, participants were asked to spend the great majority of the daytime hours (i.e., approximately 7 AM to 5 PM) inside their apartments, and their compliance was tracked via daily surveys. The study protocol is shown in Figure 2.

### 2.5. Statistical Analyses

All statistical analyses were performed using R (version 4.0.2, R Project for Statistical Computing, Vienna, Austria), and results with a *p*-value less than or equal to 0.05 were considered statistically significant. For all analyses, the within-subjects fixed factor condition comprised of two levels (EC Glass, Blinds). DLMO and actigraph-based sleep metrics were analyzed using generalized linear mixed effects models, controlling for the lifestyle factors identified a priori that were collected in the daily surveys: caffeine intake after 12 PM, evening exercise duration, evening screen time, evening alcohol intake, and evening medication use (Equation (1)). Results of the environmental measurements, phasor analysis, vitality scales, and PROMIS scores were analyzed using *t*-test comparisons of means. Finally, to assess the relationship between daytime illuminance and study outcomes, daytime illuminance measures were categorized into an ordinal measure (<100 lx, 100–300 lx, >300 lx) and DLMO, sleep onset latency, sleep duration, sleep efficiency, and sleep latency were evaluated across this ordinal variable using Kendall’s coefficiency of rank correlation.

Outcome~β_0_ + β_1_∙Condition + β_2_∙Caffeine after noon + β_3_∙Exercise + β_4_∙Screen + β_5_∙Alcohol + β_6_∙Medication + *e_i j_* + *u_i_* (1)

Distributions for each outcome measure were assessed for outliers and normality. No statistically significant far outliers (Criterion: *y* > *Q*_3_ + 3.0 × IQR, or, *y* < *Q*_1_ − 3.0 × IQR—where y is the data point, *Q*_1_ is the lower quartile, *Q*_3_ is the upper quartile, IQR is the inter-quartile range or *Q*_3_–*Q*_1_) were detected. Data were not found to be highly skewed (skewness less than −1 or greater than 1) for any of the outcome measures.

## 3. Results

### 3.1. Study Population

In total, 63 individuals completed the eligibility survey distributed throughout the apartment complex. Of those, 55 fit all eligibility criteria (age, no diagnosis of sleep conditions, working from home or otherwise home most of the day) and these 55 individuals attended remote information sessions with the researchers to discuss study participation. Of the 55, 26 maintained an interest in participating and filled out consent forms. The final 20 participants were selected based on a first-come, first-serve basis. Three participants dropped out after the first week (baseline) and were replaced immediately, maintaining 20 total participants who underwent both intervention weeks.

### 3.2. Environmental Conditions

Environmental conditions across the two intervention periods were largely similar except for daytime light levels (Table 2). Daytime illuminance measurements measured adjacent to the window and by participants’ Daysimeters revealed subtle but significant differences across the two intervention periods. A two-tailed *t*-test revealed that illuminance levels adjacent to the window were significantly higher in the EC Glass condition than the Blinds condition, with a mean daytime illuminance of 185 lx for the EC Glass condition and 148 lx for the Blinds condition (*t* = −7.76, *p* < 0.001). Various measures of circadian-effective lighting indicated higher levels the EC Glass condition compared to the Blinds condition, as reported as daytime circadian stimulus (CS) [22] (CS_EC_ = 0.156, CS_Blinds_ = 0.138); and daytime melanopic lux (Melanopic Lux_EC_ = 202.4 lux, Melanopic Lux_Blinds_ = 177.2 lux). Hourly light conditions over the course of the day and α-opic irradiance values of the two conditions are described further in Supplementary Tables S1 and S2.

### 3.3. Sleep Timing

**Figure 3:**
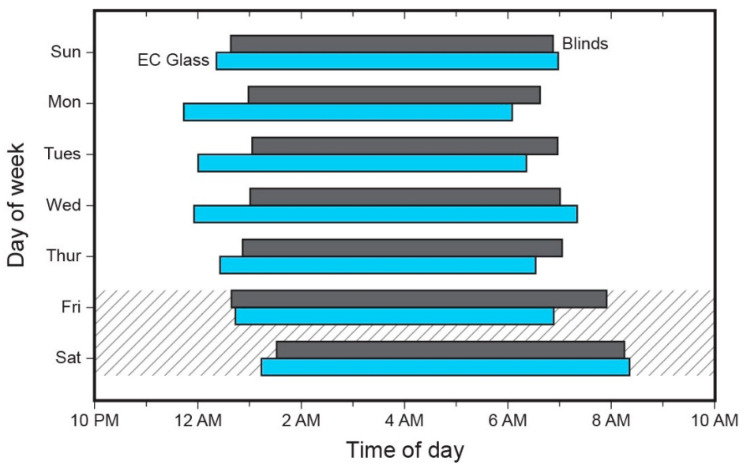
Actigraph-recorded mean sleep timing during intervention weeks, by day of week. The start of the bar (left) represents mean sleep onset time across the 20 participants, while the end of the bar (right) represents the mean wake time. The cross-hatched area represents weekend night sleep periods.

Participants exhibited later sleep onset during the blinds condition compared to the EC glass condition on weekday nights (Figure 3). Adjusting for lifestyle sleep predictors such as caffeine consumption and exercise in a within-subjects model, the participants’ mean sleep onset was 22 min later in the blinds condition compared to the EC glass condition (*p* = 0.05, Table 3). On the weekends (cross-hatched area in Figure 3), there was no difference in sleep onset timing between the two conditions. (Additional details concerning the mediating or moderating effect of caffeine are provided in the Supplementary Material; Table S3 and Figure S3.)

### 3.4. Objective Sleep Quality and Sleep Regularity

Linear mixed-effects models controlling for lifestyle sleep predictors reveal that participants exhibited significantly higher sleep regularity in the EC glass condition compared to the blinds condition (0.88-point difference, *p* = 0.001) (Table 4). No statistically significant differences in sleep duration, sleep onset latency, and sleep efficiency were revealed between the two conditions (Table 4).

**Figure 4:**
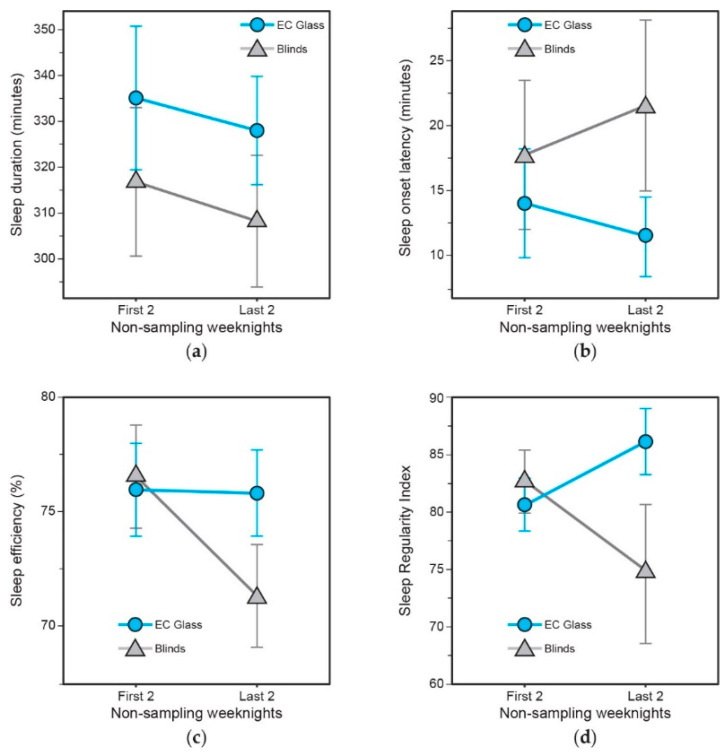
Objective sleep quality results between the two lighting conditions on the first two and last two non-sampling weeknights during the intervention periods: (**a**) sleep duration, (**b**) sleep onset latency, (**c**) sleep efficiency; and (**d**) sleep regularity (as calculated using the Sleep Regularity Index).

The differences between the start (first two non-sampling weeknights (i.e., Days 2 and 3)) and end (last two non-sampling weeknights (i.e., Days 5 and 6)) of the intervention weeks suggest a divergence in sleep onset latency, sleep efficiency and sleep regularity between the conditions with greater duration of intervention exposure (Figure 4b–d).

### 3.5. Phase Relationship between Light-Dark and Activity-Rest (Phasor Analyses)

**Figure 5:**
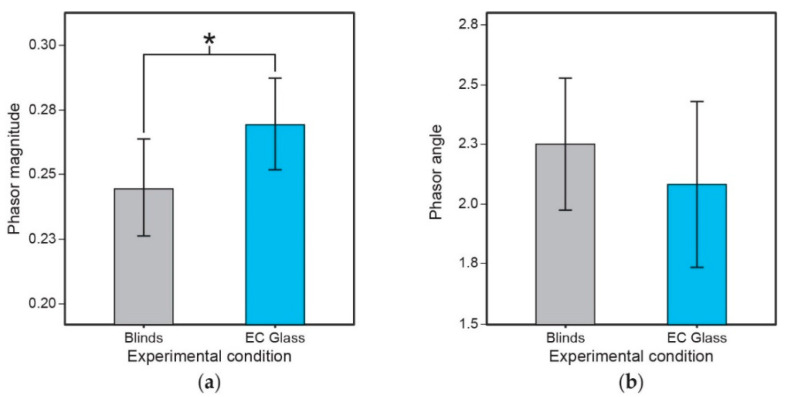
(**a**) Effect of intervention on phasor magnitude and (**b**) phasor angle as calculated from the Daysimeter data for 15 participants. The error bars represent standard error of the mean. (Statistical significance: * represents *p* ≤ 0.)

Paired sample, one-tailed t-tests revealed that phasor magnitude for the EC glass condition were significantly greater than phasor magnitude for the blinds condition (*t* = 1.91, *p* = 0.04), but no statistically significant differences were revealed for phasor angle (Figure 5). (Additional details concerning the phasor analyses results are provided in the Supplementary Material. A sample Daysimeter profile is provided in Figure S4.)

### 3.6. Dim Light Melatonin Onset (DLMO)

**Figure 6:**
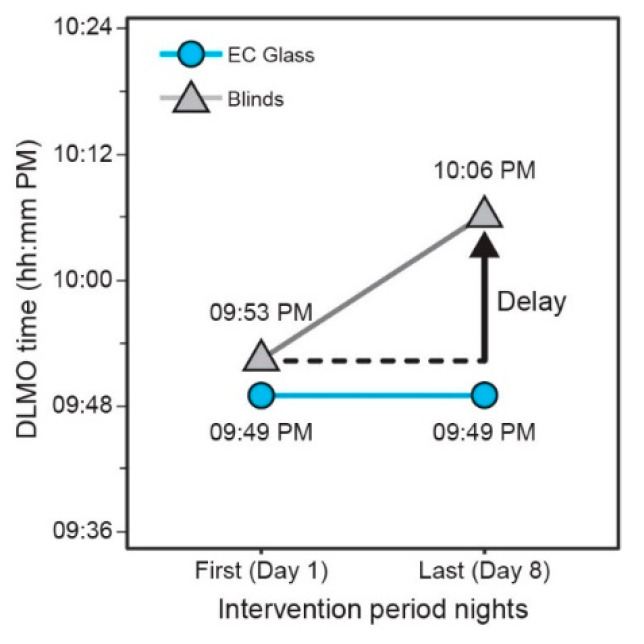
Unadjusted DLMO shift from the first (Day 1) to the last (Day 8) day of each intervention period.

While the DLMO time over the course of the EC Glass intervention period remained stable, the linear mixed-effects model revealed a delay in DLMO time over the course of the blinds intervention period. The mean DLMO Time at the start (Day 1) and end (Day 8) of the EC glass intervention period was 9:49 PM. During the blinds intervention period, however, the mean DLMO time was 9:53 PM on the first day (Day 1) and shifted to 10:06 PM on the last day (Day 8) (Figure 6). The mixed-effects model, controlling for lifestyle factors known to affect sleep and melatonin onset (i.e., caffeine intake after noon, evening exercise duration, evening screen time, evening medication use, and number of alcoholic drinks), indicated a statistically significant delay of 15 min for DLMO time in the blinds condition compared to the EC glass condition (*p* = 0.027, Table 5).

### 3.7. Subjective Mental Health and Vitality

**Figure 7:**
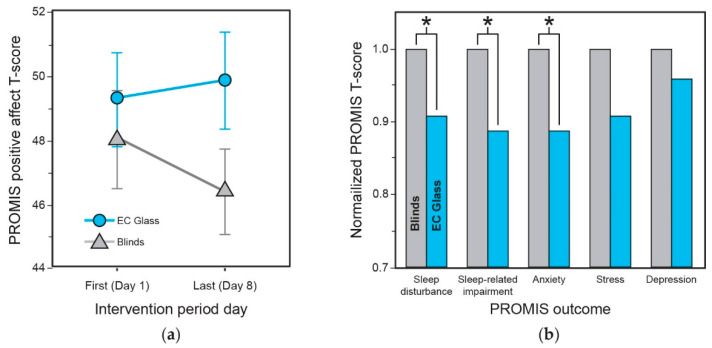
(**a**) PROMIS Positive Affect I5a instrument score (T-score) over the course of the week (start = Day 1 to end = Day 8) in the EC Glass versus Blinds conditions. (**b**) Normalized results of the linear mixed effects model comparing the PROMIS statement subscore (scale of 1–5) across interventions for sleep disturbance (*p* = 0.036), sleep-related impairment (*p* = 0.049), anxiety (*p* = 0.023), stress (*p* = 0.075), and depression (*p* = 0.30). Lower normalized PROMIS T-scores represent improved values in these outcomes. *p*-values indicate results of the within-subjects linear mixed effects model, *x*-axis describes scale statements assessed for each PROMIS instrument. (Statistical significance: * represents *p* ≤ 0.05.)

Positive affect (PROMIS Positive Affect 15a) T-scores were 3.37 points higher in the EC Glass condition compared to the Blinds condition (linear mixed effects model, +3.37 or 7% higher, *p* = 0.035) (Figure 7a). Sleep disturbance, sleep-related impairment, anxiety, stress and depression as measured weekly by the full PROMIS instruments did not yield statistically significant *t*-test results across intervention periods, likely due to the small number of observations (as it was administered only at the start and end of each period). However, mean scores for the one statement per PROMIS instrument that was administered daily demonstrated significant differences between the conditions. Within-subjects analyses for these daily statements, sleep disturbance, sleep-related impairment and anxiety as assessed by the statement subscores (on a scale of 1–5), revealed scores that were approximately 10% lower in the EC glass condition compared to the blinds condition (Figure 7b).

**Figure 8:**
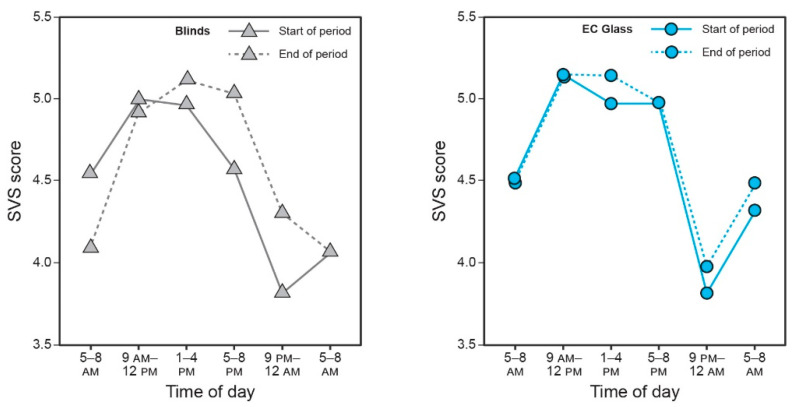
Composite SVS score across the day and following morning, at the start (Day 1) and end (Day 8) of the intervention weeks.

Self-reported vitality upon first waking (which ranged from approximately 5 AM to 8 AM across participants) as quantified by the SVS scale was significantly higher in the EC glass condition compared to the blinds condition (*t* = −2.10, *p* = 0.04). Illustrating the composite vitality (SVS) score across the intervention week demonstrates that in the EC glass condition, the timing of peak vitality did not change from the start (Day 1) to the end (Day 8) of the intervention period. In the blinds condition, on the other hand, participants exhibited high vitality immediately before bedtime, low vitality upon waking the morning after, and an overall delay in peak vitality timing over the course of the week (Figure 8).

### 3.8. Daylight and Study Outcomes

**Figure 9:**
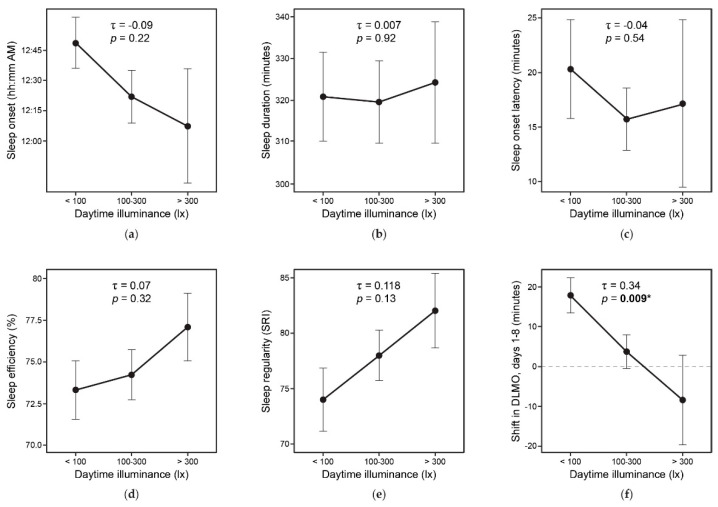
Kendall’s Rank Correlation showing the relationship between mean daytime illuminance measured in participants’ apartments and the study outcomes: (**a**) sleep onset, (**b**) sleep duration, (**c**) sleep onset latency, (**d**) sleep efficiency, (**e**) sleep regularity, and (**f**) shift in DLMO. (Abbreviations: SRI, Sleep Regularity Index; DLMO, dim light melatonin onset; statistical significance: * represents *p* ≤ 0.05.)

The relationship between indoor light conditions and study outcomes, independent of condition, are presented in Figure 9. Daytime illuminance levels measured continuously over the course of the study were categorized into low (<100 lx), medium (100–300 lx), and high (>300 lx) categories. Higher indoor light levels appear to be associated with improved circadian outcomes of earlier melatonin onset, earlier sleep times, lower sleep onset latency, longer duration, higher efficiency, and higher regularity (Figure 9). Only the correlation between melatonin onset and daytime illuminance levels was found to be statistically significant (Kendall’s Rank Correlation, τ = 0.34, *p* = 0.009).

## 4. Discussion

Over the course of this four-week, within-subjects, crossover design residential field study, all participants experienced two six-day baseline periods and two eight-day intervention periods: (1) functionally standard (i.e., clear) windows with blinds partially drawn and (2) functioning EC glass windows. It was hypothesized that the EC glass condition, designed to reduce glare and increase circadian-effective light indoors, would promote stronger alignment of endogenous rhythms (melatonin rhythm characterized by consistency in DLMO) and rest–activity patterns (characterized by phasor magnitude and sleep regularity index) with the external solar cycle, leading to improved objective and subjective sleep and mental health outcomes.

Participants exhibited a 15-min melatonin onset delay (*p* = 0.03) over the course of the intervention period when their apartments had untinted glass and blinds (i.e., the blinds condition), but a maintained consistent melatonin onset over the course of the week when the EC glass’s tinting was activated (i.e., the EC glass condition). They also demonstrated improved circadian alignment (phasor magnitude, *p* = 0.04), went to sleep 22 min earlier (*p* = 0.05), and had higher sleep regularity (*p* = 0.001) while in the EC glass condition. These impacts were likely driven by the improved daytime circadian-effective light levels imparted by the EC glass condition, supporting well-established mechanistic models demonstrating the benefits of short wavelength light characteristic of daylight on human circadian rhythm alignment and sleep quality [34]). However, in the current study, CS levels were found to be relatively low regardless of condition, an observation that could be attributed to the chest-level pendant measurements which have been previously shown to underestimate light levels reaching the eyes [35]. It is also important to note that the dynamic tuning of daylight toward shorter wavelengths by the EC glass enabled positive circadian and sleep effects of daylight at lower indoor light levels and shorter intervention length (8 days) than those reported previously [36]. Regardless, the results are in line with previous field studies investigating the impact of access to circadian-effective lighting or to daylighting indoors on sleep, mood, and alertness [6,7,37,38].

The observed DLMO and sleep delays in the Blinds condition over the course of just eight days demonstrate the sensitivity with which the human body responds to the external cues of light or daylight. These delays observed in the study, if accumulated, could lead to further curtailed total sleep given social and work lives that necessitates waking up at a fixed time. In fact, these data support the work by Roenneberg and Merrow, who showed that one needs to be exposed to at least 2 h of daylight per day to avoid experiencing social jet lag [39]. In addition, the fact that longer rapid eye movement (REM) periods occur in the later part of the sleep cycle [40] suggests that curtailing sleep in the fashion observed in the current study may lead to less REM sleep. REM sleep has been shown to be crucial for learning, cognitive and social processing, and higher-level thinking implicated in creative problem solving [41]. Previous research has found that greater daylight exposure in an office setting increases sleep duration at night and, in turn, improves decision-making performance, with test scores on 1.5 h cognitive simulations increasing by 79% on average after five days of exposure [42].

Providing the optimal light conditions indoors not only impacts circadian phase and sleep but also impacts daytime energy levels and mental health by promoting an acute alerting effect, wakefulness, and vitality [43]. In the current study, while in the EC glass condition, participants exhibited higher positive affect (*p* = 0.035) and demonstrated a distinct cycle of high vitality throughout the day, low energy at night, and high vitality again after waking the next morning, a cycle that remained relatively consistent from the start to end of the week. Meanwhile, in the blinds condition, they exhibited a delay in peak vitality, higher nighttime energy levels, and lower morning vitality at the end of the week compared to the start. These results are consistent with those from Figueiro and colleagues, showing that subjective vitality increased after exposure to circadian-effective light during the day [32].

Implementing proper circadian-effective lighting indoors is particularly important given the downstream chronic health consequences of sustained circadian and sleep disruption. Circadian misalignment has been implicated in cardiovascular disease by increasing blood pressure and inflammatory mediators and reducing heart rate recovery [44,45]; metabolic diseases such as diabetes, obesity, and insulin resistance [46,47,48]; Alzheimer’s disease [46]; and impaired cognitive performance [49]. Furthermore, evidence from studies focused on jet lag, night-shift work, and seasonal affective disorder suggest strong associations between chronic circadian misalignment and increased precipitation or exacerbation of mood disorders such as depression, anxiety, and bipolar disorder [50,51,52]. More recently, day-to-day regularity of the sleep schedule has received particular attention for its association with poor mood and depression, irrespective of total time spent asleep [53]. Moreover, older adults with irregular sleep patterns have been found nearly twice as likely to develop cardiovascular disease and metabolic abnormalities [54,55], and college students with irregular sleep were found to have poorer academic performance [19]. Chronic circadian misalignment has also been implicated in cancers of the breast, prostate, and colon [56]. In fact, the International Agency for Research on Cancer (IARC) recently categorized nighttime shift work as a probable human carcinogen [57]. The mechanisms behind these health conditions have been extensively studied in model organisms bred with and without proper circadian function or exposed to circadian-disrupting cues [58,59,60].

The weeklong interventions investigated in the current study limits the ability to extrapolate the results to longer exposures. However, previous research suggests that phase shifts may be additive in the continued absence of sufficient circadian-effective daytime light exposure [61,62]. In addition, most of the study population was healthy and young, limiting the generalizability of these findings and implications to other populations. For example, those who suffer from sleep impairments or Alzheimer’s disease have been shown to benefit from exposure to daytime circadian-effective light [63], and may benefit to an even greater degree than healthy young adults due to the amount of room for improvement. The study was also limited in that the participants were not given any instruction as to the use of electric lighting during the study (except during DLMO collection). However, this was a random factor that was common to both conditions and reflects the realistic nature of the experimental design. Lastly, the current study focused only on two experimental conditions, one with functionally standard glass and blinds and one with dynamically tinting EC glass; other window façade configurations were not studied.

The within-subjects, balanced crossover study design with washout periods conferred many strengths to the study. The balanced crossover design ensured that the results were not an artifact of temporal factors or learning effects, and the within-subjects design allowed each participant to serve as their own control. Furthermore, the study utilized objective measures (the Daysimeter, salivary melatonin, and actigraphy) to reproduce the mechanistic pathway from daylight exposures to hormone levels, sleep behaviors, and daytime vitality. The greatest strength of the study, however, was the face validity of the study setting and interventions. This field study was conducted in a real-world environment, with participants living in their own homes experiencing a realistic set of daylight exposures for one week at a time without intervening in their lifestyle or behaviors.

## 5. Conclusions

This study found that residents (healthy adults from the general population) demonstrated greater circadian alignment, earlier and more regular sleep, and improved vitality and mental health when living in an apartment with EC glass than one with traditional glass and blinds. Given the critical role of light on the human circadian system, the downstream consequences of circadian misalignment on mental and physical health, and the fact that humans spend nearly 90% of their time indoors [64], it is critical to incorporate daylighting solutions in buildings that go beyond meeting visual task requirements and promote circadian alignment and health more broadly. This study demonstrates one successful method of implementing more daylight in the built environment and presents the opportunity that EC glass technology has for optimizing indoor daylight access and optimizing occupant health without the drawbacks of visual and thermal discomfort, energy consumption, and reliance on occupant behaviors that come with traditional façade solutions. The study also demonstrated that designing for daylight is not only beneficial for buildings that house the sick, but also imparts substantial health benefits for the general population. These benefits of daylight should be considered by developers and architects when designing buildings intended to be human-centered.

## Figures and Tables

**Table 1:** Demographic and chronotype data collected for the 20 study participants.

Attribute	Variable	n
Sex	Female	11 (55%)
	Male	8 (40%)
	Non-binary	1 (5%)
Age	Mean (SD)	35 (13)
	Median (Min, Max)	33 (21, 77)
Race	Asian	2 (10%)
	Black	3 (15%)
	Hispanic/Latino/Spanish	3 (15%)
	Multiracial	3 (15%)
	White	9 (45%)
Education	High school graduate	2 (10%)
	Some college	1 (5%)
	College degree	9 (45%)
	Graduate degree	8 (40%)
Income	$25 K–$75 K	2 (10%)
	$75–$100 K	4 (20%)
	$100–$125 K	4 (20%)
	$125–$150 K	2 (10%)
	>$150 K	5 (25%)
	Prefer not to answer	3 (15%)
Employment	No	5 (25%)
	Yes	15 (75%)
General health	Poor	—
	Fair	1 (5%)
	Good	4 (20%)
	Very good	11 (55%)
	Excellent	4 (20%)
Medication use	Allergy	4 (20%)
	Anxiety	—
	Melatonin	—
	Sleep	—
MCTQ ^a^ sleep duration (workday)	Mean hours (SD)	8.2 (0.9)
	Median hours (Min, Max)	8.3 (5.8, 9.5)
MCTQ ^a^ sleep duration (non-workday)	Mean hours (SD)	8.1 (1.3)
	Median hours (Min, Max)	8.2 h (6.0, 12)
MCTQ score ^b^	Mean (SD)	4.1 (1.0)
	Median (Min, Max)	4.0 (2.0, 5.0)
	Slightly early	2 (10%)
	Normal	3 (15%)
	Slightly late	7 (35%)
	Moderately late	8 (40%)

Notes: ^a^ MCTQ = Munich ChronoType Questionnaire, ^b^ Chronotype score ranges from zero (extremely early) to 6 (extremely late).

**Table 2:** Mean and median daytime environmental conditions across study conditions. All values reflect daytime means or medians, as defined by sunrise (6:56 AM) to sunset (4:52 PM). Air quality, thermal conditions, noise levels, and illuminance were collected by the Awair Omni devices located on the wall of the living room. Daytime circadian stimulus was collected at participant chest-level using Daysimeter devices and spectrally weighted melanopic lux was derived from calibrated Daysimeter RGB sensor data.

Condition	Parameter (Unit)	Daytime Mean (7 AM to 5 PM)		Daytime Median (7 AM to 5 PM)	
		Blinds	EC Glass	Blinds	EC Glass
Light	Illuminance (lx)	148.7	185.3	66.2	115.7
	Circadian stimulus	0.138	0.156	0.105	0.127
	Melanopic lux	177.2	202.4	129.8	253.1
Air Quality	CO_2_ (ppm)	795.1	776.4	695.2	670.4
	PM2.5 (ug/m^3^)	18.4	17.5	2.9	3.3
Thermal	Temperature (°F)	70.6	71.1	70.2	71.0
	Relative humidity (%)	37.9	39.3	37.	38.6
Noise	Noise (dB)	55.6	55.3	54.6	54.1

**Table 3:** Results of the linear mixed effects model of intervention and weekday sleep onset timing.

**Predictor**	**Difference in Sleep Onset Time (Minutes)**	
	**Estimate**	***p***
Intercept	12:33 AM	0.215
Condition (EC Glass)	−22.2	0.050
Caffeine after 12 PM (yes)	−4.8	0.790
Exercise after 5 PM (hours)	−7.2	0.788
Alcoholic drinks (number)	6.0	0.344
Medication after 5 PM (yes)	6.6	0.774
Screen duration after 5 PM (hours)	1.8	0.718

**Table 4:** Results of the linear mixed effects models for actigraph-measured sleep quality metrics between the two experimental conditions, controlling for lifestyle factors.

Predictor	Duration (min)		Efficiency (%)		Latency (min)		Regularity (SRI)	
	Estimate	*p*-Value	Estimate	*p*-Value	Estimate	*p*-Value	Estimate	*p*-Value
Intercept	318.18	<0.001	72.47	<0.001	18.86	0.003	81.63	<0.001
Condition (EC Glass)	16.11	0.169	0.42	0.821	−5.12	0.319	0.88	0.001
Caffeine after 12 PM (yes)	−12.50	0.450	−0.44	0.868	−1.48	0.806	1.54	<0.001
Exercise after 5 PM (hours)	42.71	0.113	−0.36	0.933	13.25	0.239	−6.09	<0.001
Alcoholic drinks (n)	−5.57	0.324	0.47	0.606	−1.70	0.402	−0.04	0.722
Medication after 5 PM (yes)	−4.20	0.823	−1.12	0.717	1.65	0.792	−4.23	<0.001
Screen duration after 5 PM (hours)	−0.40	0.934	0.52	0.499	0.51	0.745	−0.59	<0.001

**Table 5:** Results of the linear mixed-effects model of condition and dim light melatonin onset.

	DLMO Onset Shift (Minutes)	
Predictor	Estimate (95% CI)	*p*
Intercept	−16.63	<0.001
Condition (EC Glass)	−15.07	0.027
Caffeine after 12 PM (yes)	−3.95	0.317
Exercise after 5 PM (hours)	2.27	0.860
Alcoholic drinks (n)	2.13	0.675
Medication after 5 PM (yes)	4.04	0.614
Screen duration after 5 PM (hours)	1.43	0.486

## Data Availability

The data that support the findings of this study are available from the authors upon reasonable request.

## Supplementary Materials

The following are available online at https://www.mdpi.com/article/10.3390/ijerph18199980/s1, Figure S1: Relative spectral power distributions (SPDs) for a daylight D65 source depicting changes in spectral profile post attenuation by four pre-programmed tint states as experienced by the occupants, Figure S2: (a) Daily illuminance over the course of the day in each of the two intervention weeks, as measured by Awair Omni devices mounted on participant’s living room wall; (b) Circadian stimulus over the course of the day collected at participant chest-level, as measured by Daysimeter devices using calibrated RGB sensors; (c) Spectrally weighted melanopic illuminance (melanopic lux) values over the course of the day collected at participant chest-level, as measured by Daysimeter devices using calibrated RGB sensors, Table S1: The α-opic irradiances for both experimental conditions calculated using the CIE S 026 α-opic Toolbox (v1.049) [1] (based upon means), Table S2: The α-opic irradiances for both experimental conditions calculated using the CIE S 026 α-opic Toolbox (v1.049) [1] (based upon medians), Figure S3: Mean caffeine intake (a) and timing (b) between the two conditions, Table S3: Causal mediation analysis for the role of caffeine timing on the pathway of intervention and circadian outcomes, Figure S4: Daysigram depicting the circadian light exposure and activity profile over the course of the 1-week data collection period for a representative participant experiencing the EC Glass condition.
