# Supplementary material for: Access to Daylight at Home Improves Circadian Alignment, Sleep, and Mental Health in Healthy Adults: A Crossover Study
Source: Int J Environ Res Public Health. 2021 Sep 23;18(19):9980. doi: 10.3390/ijerph18199980 (PMC8507741; doi:10.3390/ijerph18199980)
Supplement: Supplementary file 1 [file ijerph-18-09980-s001.zip › ijerph-1265187-supplementary.pdf]

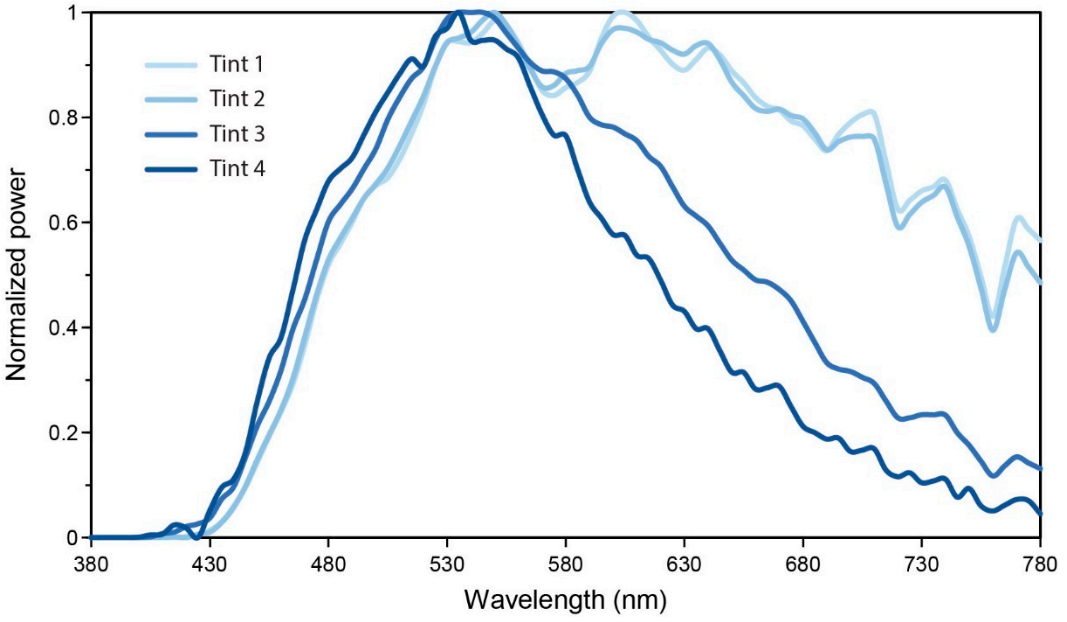

Figure S1. Relative spectral power distributions (SPDs) for a daylight D65 source depicting changes in spectral profile post attenuation by four pre-programmed tint states as experienced by the occupants.

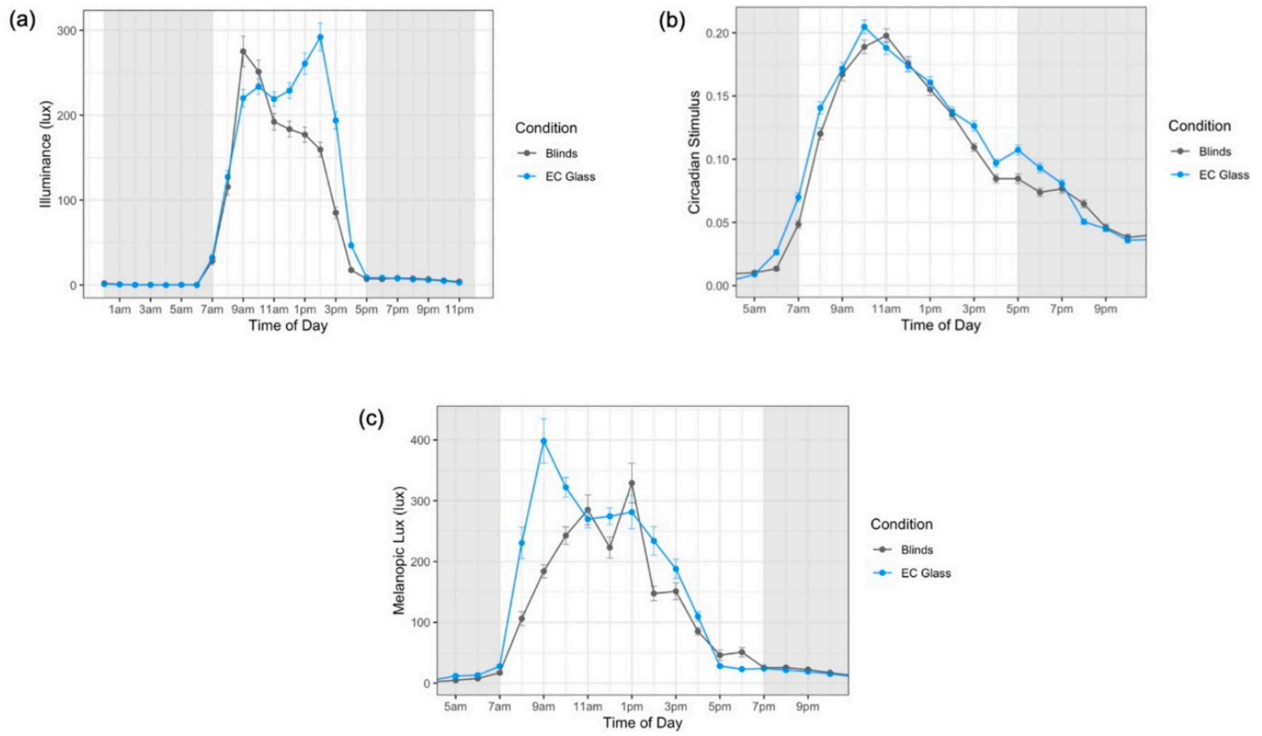

Figure S2. (**a**) Daily illuminance over the course of the day in each of the two intervention weeks, as measured by Awair Omni devices mounted on participant's living room wall; (**b**) Circadian stimulus over the course of the day collected at participant chest-level, as measured by Daysimeter devices using calibrated RGB sensors; (**c**) Spectrally weighted melanopic illuminance (melanopic lux) values over the course of the day collected at participant chest-level, as measured by Daysimeter devices using calibrated RGB sensors.

α-opic Irradiances for Experimental Conditions

Daylight D65 was modelled as the baseline spectrum for all α-opic irradiance calculations. The baseline D65 was subjected to a time-weighted tint attenuation profile and the output absolute spectral power distribution, at the average photopic light levels recorded using the Daysimeter devices, was provided as input the CIE S 026 α-opic toolbox to calculate following α-opic irradiances.

Table S1. The α-opic irradiances for both experimental conditions calculated using the CIE S 026 α-opic Toolbox (v1.049) [1] (based upon means).

Stimulus Lights	S-cone-opic irradiance W m^{-2}	M-cone-opic irradiance W m^{-2}	L-cone-opic irradiance W m^{-2}	Rhodopic irradiance W m^{-2}	Melanopic irradiance W m^{-2}
Electrochromic glass (Daylight D65 at 148.7 lx) Tint 1 (**"Blinds"**)	0.02	0.19	0.24	0.14	0.11
Electrochromic glass (Daylight D65 at 185.3 lx) Dynamic tint ("**EC Glass**")	0.03	0.24	0.30	0.19	0.15

Table S2. The α-opic irradiances for both experimental conditions calculated using the CIE S 026 α-opic Toolbox (v1.049) [1] (based upon medians).

Stimulus Lights	S-cone-opic irradiance W m^{-2}	M-cone-opic irradiance W m^{-2}	L-cone-opic irradiance W m^{-2}	Rhodopic irradiance W m^{-2}	Melanopic irradiance W m^{-2}
Electrochromic glass (Daylight D65 at 66.2lx) Tint 1 ("**Blinds**")	0.01	0.09	0.11	0.06	0.05
Electrochromic glass (Daylight D65 at 115.7 lx) Dynamic tint ("**EC Glass**")	0.02	0.15	0.19	0.12	0.09

Phasor Analyses Results

Paired sample one-tailed t-test revealed that the phasor magnitudes (PM) for the EC Glass condition were significantly greater than the phasor magnitudes for the Blinds condition (t_{14} = 1.91, $p < 0.05$), suggesting a greater synchrony between the light-dark cycle and the rest-activity patterns among participants for the EC Glass condition. The mean phasor magnitudes for the EC Glass and Blinds condition were 0.27 and 0.24, respectively. Overall, the degree of circadian alignment for participants under both conditions was noticeably lower compared to that reported in previous studies for a healthy older adult (PM = 0.40 [5]), or a day-shift working nurse (PM = 0.42 [6]). The phasor magnitudes for both the conditions were marginally higher compared to those previously reported for office workers experiencing low morning CS levels (PM = 0.23 [7]), and substantially higher than those previously reported for rotating-shift working nurses (PM = 0.13 [6]).

Paired sample one-tailed t-test revealed that phasor angles (PA) for the EC Glass condition were not significantly smaller than the phasor angles for the Blinds condition (t_{14} = -0.76, p = 0.22), suggesting a similar circadian phase for the daily onset of activity for participants across both the interventions. The mean phasor angles for the EC Glass and the Blinds condition were 2.08 and 2.25, respectively. Overall, the circadian phase for participants under both the conditions was noticeably delayed compared to that reported in previous studies for a healthy older adult (PA = 1.31 [5]), or a day-shift working nurse (PA = 0.81 [6]), or an office worker receiving high morning CS (PA = 1.04 [7]). The phasor angles for both the conditions were modestly advanced compared to those previously reported for rotating-shift working nurses (PA = 2.41 [6])

The low phasor magnitudes and delayed phasor angles suggest that the study participants were of peculiarly late chronotypes, which is also reflected in late sleep onset times recorded using actigraphs.

Mediating or Moderating Effect of Caffeine

Lifestyle factors as collected in the daily surveys were assessed for potential mediation of the association between intervention and circadian outcomes. Number of caffeinated drinks consumed on intervention days, evening screen exposure duration, evening exercise duration, and evening alcohol consumption, and melatonin use (none, across all participants and days) were consistent across intervention weeks. Timing of caffeine consumption, however, appeared to differ. While 34% of participants consumed caffeine after 12 PM in the Blinds condition, the rate was 23% in the EC Glass condition (Figure S4). Potential mediation of caffeine timing was assessed using bootstrap analysis (R package

‘mediation) and found that it was not a significant mediator of the relationships between intervention and sleep onset timing, duration, efficiency, nor latency (Table S3). Potential effect modification by caffeine timing was also assessed but found to be non-significant (Figure S3).

Table S3. Causal mediation analysis for the role of caffeine timing on the pathway of intervention and circadian outcomes.

Sleep Outcome	Effect of Intervention (EC Glass)[a]		Mediation by Caffeine[b]	
	β	p-value	Proportion mediated	*p*-value
Sleep onset (minutes)	-22	0.05	1.4%	0.724
Sleep duration (minutes)	16	0.17	4%	0.66
Sleep efficiency (%)	0.4	0.821	<1%	0.98
Sleep onset latency (minutes)	-5	0.319	<1%	0.92

[a] Unadjusted linear mixed effects model results for the effect of Condition (EC Glass vs. Blinds) on sleep onset, duration, efficiency, and latency. [b] Caffeine intake after 12pm, a binary variable collected via daily surveys, was assessed as a potential mediator between intervention and sleep outcomes using bootstrap analysis.

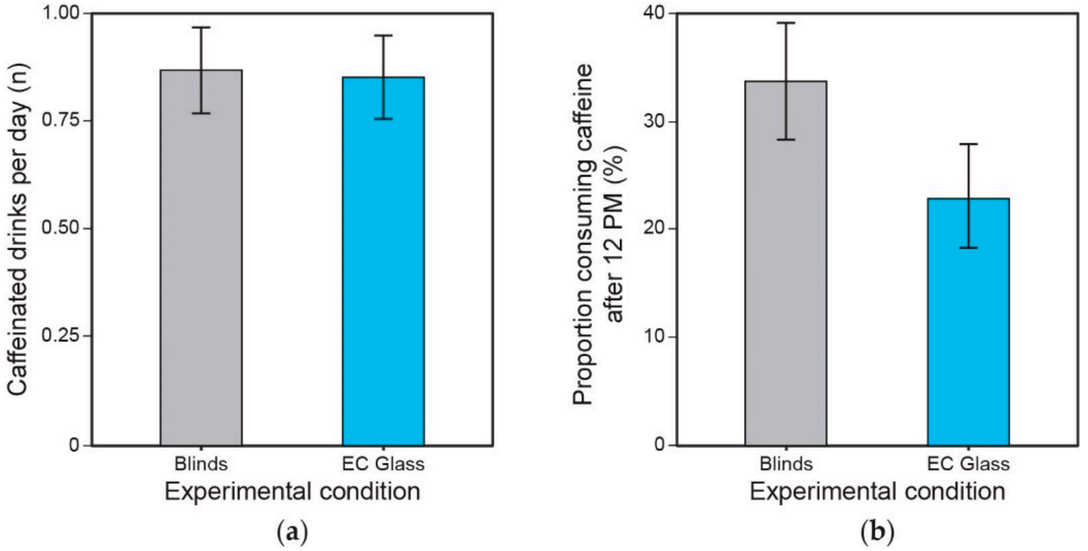

Figure S3. Mean caffeine intake (**a**) and timing (**b**) between the two conditions.

Daysimeter and Phasor Information

The Daysimeter devices employed in this study were developed and calibrated by the Lighting Research Center, Rensselaer Polytechnic Institute, to measure and record personal circadian light exposure (CL_A) [2,3] and activity levels, continuously over several days. It comprises of a solid-state accelerometer package and a RGB solid-state photosensor package with a cosine spatial distribution similar to the human eye [4]. The optical radiation and activity data recorded by these Daysimeter devices were downloaded onto a secure computer using the freely available Daysimeter 12 GUI Python client (V1.1.10, 2017, Lighting Research Center, Troy, NY).

The 24-hour phasor magnitude is used as the metric for behavioral circadian entrainment/disruption, wherein greater the magnitude (range: 0–0.7), the greater the level of behavioral circadian alignment of activity to light. The phasor angle (range: +12 to -12 h) reflects the phase relationship between the periodic light-dark exposure pattern and the periodic activity-rest pattern in the correlations. To perform a reliable circular correlation of the light and activity time series, minimum 3 days of clean data is required. Preliminary analysis of light exposure/activity profiles for all study participants, revealed that 5 participants failed to meet the threshold criteria of at least 3 days of Daysimeter compliance and hence could not be included in the phasor analysis. A sample Daysimeter profile (“Daysigram”) depicting light and activity data across one week of data is illustrated in Figure S3.

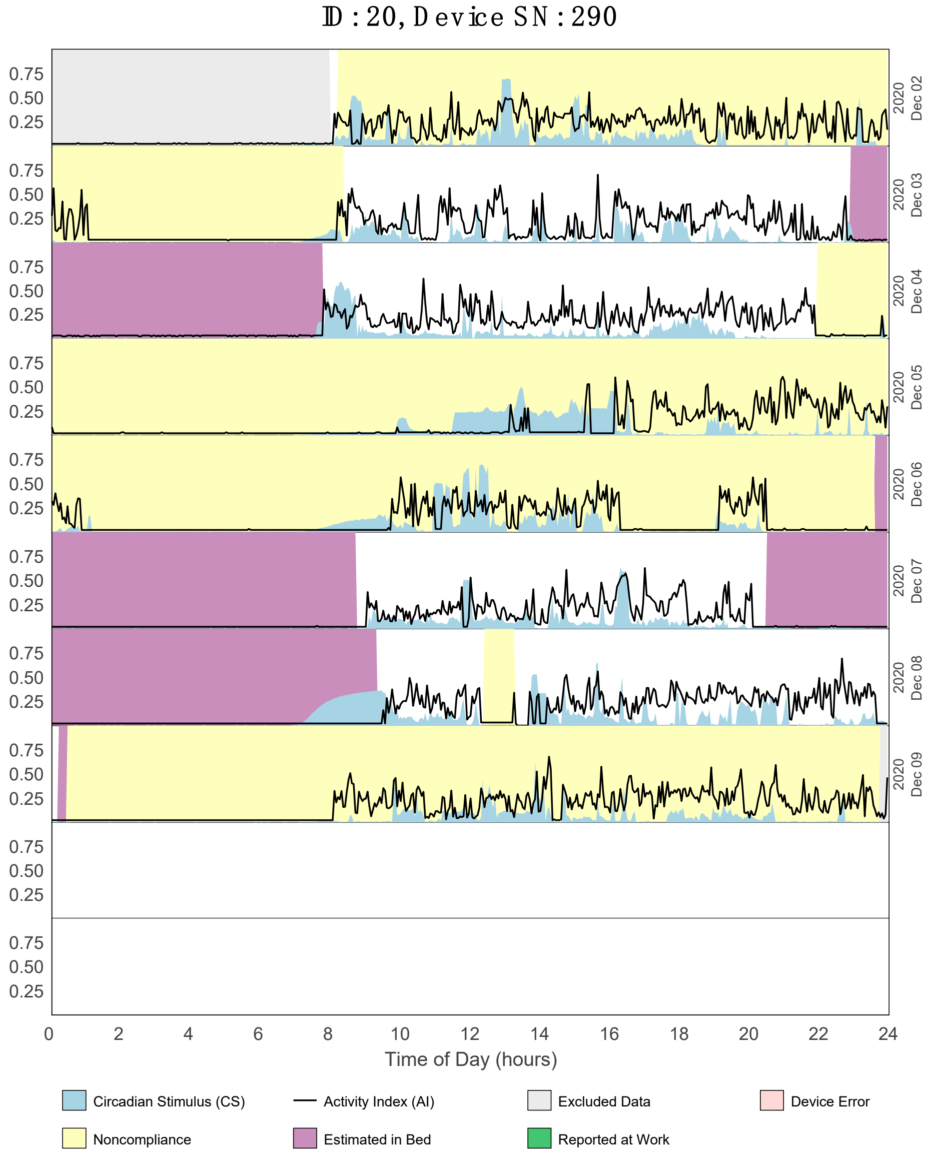

Figure S4. Daysigram depicting the circadian light exposure (blue) and activity profile (black) over the course of the 1-week data collection period for a representative participant experiencing the EC Glass condition. The data highlighted in yellow comprises of non-compliance, as well as data excluded from weekends and biospecimen sampling days.

References

1. Commission Internationale de l'Éclairage *The α-opic Toolbox*, v1.049, 2020/03/26; Commission Internationale de l'Éclairage: Vienna, 2020.
2. Rea, M.S.; Figueiro, M.G.; Bullough, J.D.; Bierman, A. A model of phototransduction by the human circadian system. *Brain Res. Rev.* **2005**, *50*, 213-228, doi:10.1016/j.brainresrev.2005.07.002.
3. Rea, M.S.; Figueiro, M.G.; Bierman, A.; Hamner, R. Modelling the spectral sensitivity of the human circadian system. *Lighting Res. Technol.* **2012**, *44*, 386-396, doi:10.1177/1477153511430474.
4. Bierman, A.; Klein, T.R.; Rea, M.S. The Daysimeter: A device for measuring optical radiation as a stimulus for the human circadian system. *Meas. Sci. Technol.* **2005**, *16*, 2292-2299, doi:10.1088/0957-0233/16/11/023.
5. Figueiro, M.G.; Hamner, R.; Higgins, P.; Hornick, T.; Rea, M.S. Field measurements of light exposures and circadian disruption in two populations of older adults. *Journal of Alzheimer's Disease* **2012**, *31*, 711-715, doi:10.3233/JAD-2012-120484.
6. Rea, M.S.; Bierman, A.; Figueiro, M.G.; Bullough, J.D. A new approach to understanding the impact of circadian disruption on human health. *J. Circadian Rhythms* **2008**, *6*, 7, doi:10.1186/1740-3391-6-7.
7. Figueiro, M.G.; Steverson, B.; Heerwagen, J.; Kampschroer, K.; Hunter, C.M.; Gonzales, K.; Plitnick, B.; Rea, M.S. The impact of daytime light exposures on sleep and mood in office workers. *Sleep Health* **2017**, *3*, 204-215, doi:10.1016/j.sleh.2017.03.005.
